# The pathological roles of intrinsically disordered proteins in lysosomal acidification dysfunction in neurodegenerative diseases

**DOI:** 10.1186/s40035-026-00591-z

**Published:** 2026-10-28

**Authors:** Kholoud Abd-ElRaouf, Dylan T. Murphy, Lenny Yi Tong Cheong, Jialiu Zeng, Chih Hung Lo

**Affiliations:** 1 Department of Biology, Syracuse University, Syracuse, NY 13244 USA; 2 Interdisciplinary Neuroscience Program, Syracuse University, Syracuse, NY 13244 USA; 3 School of Biological Sciences, Nanyang Technological University, Singapore, Singapore, 637551 Singapore; 4 Department of Biomedical and Chemical Engineering, Syracuse University, Syracuse, NY 13244 USA

**Keywords:** Intrinsically disordered proteins, Lysosomal acidification, Autophagy, Protein degradation, Neuroinflammation and neurodegeneration, Therapeutic strategies

## Abstract

Neurodegenerative diseases such as Alzheimer’s disease (AD), frontotemporal dementia (FTD), and Parkinson’s disease (PD) are characterized by the accumulation of intrinsically disordered proteins (IDPs), including amyloid-β (Aβ), tau, and α-synuclein (αSyn). These proteins disrupt neuronal homeostasis through toxic aggregation, impaired proteostasis, and progressive neurodegeneration, in part via dysfunction of the autophagy-lysosomal pathway (ALP). A central feature of this impairment is defective vacuolar-type (H^+^)-ATPase (V-ATPase) function, leading to lysosomal deacidification and reduced degradative capacity. Pathological IDP species converge on V-ATPase through direct interactions with its subunits or by perturbing its assembly, while also exerting protein-specific effects, including lysosomal membrane destabilization, impaired hydrolase activity, and disrupted endolysosomal trafficking. These combined mechanisms create a reciprocal relationship between lysosomal dysfunction and IDP accumulation, in which impaired lysosomal degradation further promotes the persistence and aggregation of pathological IDP species. This review synthesizes current insights into the molecular interplay between IDPs and lysosomal acidification across AD, FTD, and PD, and highlights how these processes contribute to disease progression. We further discuss emerging therapeutic strategies aimed at restoring lysosomal function. These findings underscore lysosomal dysfunction as a common feature of IDP-driven neurodegeneration and a potential therapeutic target across these disorders.

## Introduction

Alzheimer’s disease (AD), frontotemporal dementia (FTD), and Parkinson’s disease (PD) are neurodegenerative disorders characterized by cognitive, behavioral, and/or motor deficits. Despite their clinical heterogeneity, they converge on a common molecular pathology: the misfolding and aggregation of intrinsically disordered proteins (IDPs). Key examples include amyloid-β (Aβ), tau, and α-synuclein (αSyn), which form amyloid plaques, neurofibrillary tangles (NFTs), and Lewy bodies, respectively, and frequently exhibit pathological co-occurrence and crosstalk across disease contexts [1–4]. Misfolded IDPs drive neurotoxicity through both loss of native function and toxic gain-of-function mechanisms, particularly via the formation of soluble oligomers and fibrillar aggregates that disrupt cellular homeostasis, including impairments in lysosomal trafficking, endolysosomal transport, and degradative clearance pathways [1–5]. Key contributors to toxic IDP accumulation include impaired autophagosome biogenesis, defective lysosomal acidification, and disrupted autophagic flux, collectively leading to impaired proteostatic clearance and neuronal death [6, 7].

### Autophagy-lysosomal pathway and vacuolar-type (H^+^)-ATPase (V-ATPase)-mediated lysosomal acidification in neurons

Autophagy and lysosomal pathways are essential for maintaining cellular homeostasis by degrading and recycling damaged organelles, misfolded proteins, and toxic aggregates, with lysosomes providing the acid hydrolases required for intracellular cargo breakdown [8, 9]. Neurons are particularly susceptible to the accumulation of toxic protein species, highlighting their dependence on an efficient autophagy-lysosomal pathway (ALP) to preserve cellular integrity. Emerging evidence suggests that aberrant processing of amyloid precursor protein (APP) and tau can disrupt V-ATPase function, leading to impaired lysosomal acidification and reduced degradative capacity. These pathogenic interactions between IDPs and V-ATPase promote the accumulation of toxic aggregates and contribute to neuroinflammation and neuronal injury [7, 10]. Overall, these findings underscore lysosomal dysfunction as a central feature of neurodegenerative diseases and reinforce the ALP as a key therapeutic target [8, 9].

Maintaining a balance between the production and the clearance of toxic protein species is critical for neuronal health. Efficient lysosomal degradation requires an acidic luminal pH (approximately 4.5–5.0), which is primarily established by the V-ATPase complex and its associated regulators [8, 11]. Lysosomes fuse with endosomes containing substrates such as IDP aggregates, exposing them to hydrolytic enzymes, including cathepsins, that depend on this acidic environment for optimal proteolytic activity. Disruption of lysosomal pH compromises enzyme function and impairs substrate clearance. Notably, excessive acidification can also be detrimental. Dysregulation of key modulators, including N-deacetylase/N-sulfotransferase 3 (NDST3), transmembrane protein 175 (TMEM175), and solute carrier family 7 member 11 (SLC7A11), has been linked to lysosomal hyperacidification, which in turn perturbs hydrolase activity and reduces protein degradation efficiency [12–14]. These observations highlight the importance of maintaining lysosomal pH within a narrow optimal range to support proper enzymatic function. Collectively, the V-ATPase-dependent proton transport is central to lysosomal function and to the clearance of toxic IDPs implicated in neurodegenerative disease.

The mammalian V-ATPase is a rotary ATP-dependent proton pump essential for intracellular pH regulation, lysosomal acidification, and organelle function. It consists of two functional domains: the cytosolic V1 sector, which hydrolyzes ATP, and the membrane-embedded V0 sector, which mediates proton translocation. The V1 domain is composed of eight subunits (A–H), whereas the V0 domain includes multiple subunits (a, c, c′, c″, d, and e) that together form the proton-conducting pathway [15, 16]. Beyond proton pumping, V-ATPase contributes to membrane trafficking, vesicle fusion, pH sensing, and mechanistic target of rapamycin complex 1 (mTORC1) signaling. These functions are further modulated by associated proteins such as ATP6AP1 (Ac45) and ATP6AP2 (pro-renin receptor), which are important for complex assembly, stability, and subcellular localization [17, 18]. The V1 subunit ATP6V1A plays a key role in neuronal homeostasis and autophagy, as its depletion leads to accumulation of LC3-II and p62, indicative of impaired autophagic flux and increased cellular stress [19]. Similarly, components of the V0 sector, including ATP6V0C, are required for efficient lysosomal acidification and maintenance of autophagic degradation in neurons [20]. Mutations that disrupt V-ATPase structure or function can impair proton translocation and lysosomal acidification. For example, the R740Q mutation in the V0a1 subunit interferes with critical interactions required for proton movement across the membrane, while additional variants such as A505P and R804H, as well as mutations in *ATP6AP2*, compromise lysosomal function, autophagy, and synaptic vesicle loading [21, 22]. In AD, presenilin-1 dysfunction impairs N-glycosylation of the V0a1 subunit, leading to defective lysosomal targeting and reduced acidification [23]. Similarly, in amyotrophic lateral sclerosis (ALS) and FTD, dysfunction of ubiquilin proteins disrupts lysosomal function; notably, UBQLN2 is required to maintain ATP6V0A1 levels necessary for proper V-ATPase function [24]. These findings underscore the central role of V-ATPase in maintaining neuronal proteostasis and highlight its dysfunction as a key contributor to neurodegenerative disease.

Ion channels play a critical role in lysosomal acidification by regulating ionic flux across the lysosomal membrane. Proton pumping by the V-ATPase is electrogenic and generates a transmembrane potential that would otherwise limit further proton accumulation. To sustain luminal acidification, counter-ion conductance, mediated by channels and transporters such as Cl^−^ and K^+^ pathways, is required to dissipate this charge, thereby enabling efficient proton influx and supporting lysosome-dependent processes including intracellular signaling and autophagy [25–29]. Two-pore channel 2 (TPC2), a lysosomal cation channel permeable to Na^+^ and Ca^2+^, regulates endolysosomal trafficking and autophagy through ion-dependent signaling mechanisms [25]. Under nutrient deprivation, mTORC1 dissociates from the lysosomal surface, coinciding with changes in lysosomal ion dynamics that contribute to autophagic activation and autophagosome-lysosome fusion [26]. TRPML1 (MCOLN1) is a lysosomal Ca^2+^ channel that links lysosomal function to transcriptional responses by promoting Ca^2+^-dependent activation of calcineurin and subsequent nuclear translocation of transcription factor EB (TFEB), thereby coordinating lysosomal biogenesis and autophagic capacity [27]. In addition to Ca^2+^ channels, chloride and potassium transport are essential for maintaining lysosomal ion homeostasis. ClC-7, a Cl^−^/H^+^ antiporter localized to late endosomes and lysosomes, facilitates charge compensation during V-ATPase-mediated proton pumping. Loss of ClC-7 disrupts lysosomal function and leads to neurodegeneration and lysosomal storage phenotypes in mice [28]. ATP13A2, a lysosomal P5-type ATPase associated with PD, contributes to lysosomal function and proteostasis, with its dysfunction impairing degradative capacity and indirectly affecting lysosomal acidification [29]. Overall, V-ATPase activity and coordinated ion transport establish and maintain the acidic lysosomal environment required for protein quality control through autophagy and lysosomal degradation pathways.

### The role of IDPs in neurodegenerative diseases

IDPs comprise a class of proteins that lack a stable three-dimensional structure over all or part of their sequence under physiological conditions [30, 31]. IDPs exist along a spectrum, ranging from proteins containing localized intrinsically disordered regions to those that remain largely disordered throughout their entire sequence [30, 31]. Unlike proteins with well-defined conformations, IDPs adopt dynamic ensembles of structures, enabling functional versatility and interactions with multiple binding partners. However, these same structural properties also predispose them to aberrant conformational transitions and aggregation [30, 31]. IDPs are typically enriched in polar and charged residues and exhibit low hydrophobicity, features that underlie their conformational flexibility and influence their aggregation behavior, particularly under conditions of cellular stress. Aggregation is generally initiated through the self-association of monomeric proteins into soluble oligomeric intermediates, often via nucleation-dependent processes [30, 31]. Accumulating evidence suggests that these soluble oligomers are among the most toxic species in multiple neurodegenerative diseases [32–35]. Continued oligomerization can give rise to more ordered, β-sheet-rich fibrillar aggregates, which are characteristic of pathological inclusions such as Aβ plaques, tau NFTs, and αSyn-containing Lewy bodies [1, 36, 37].

**Fig. 1:**
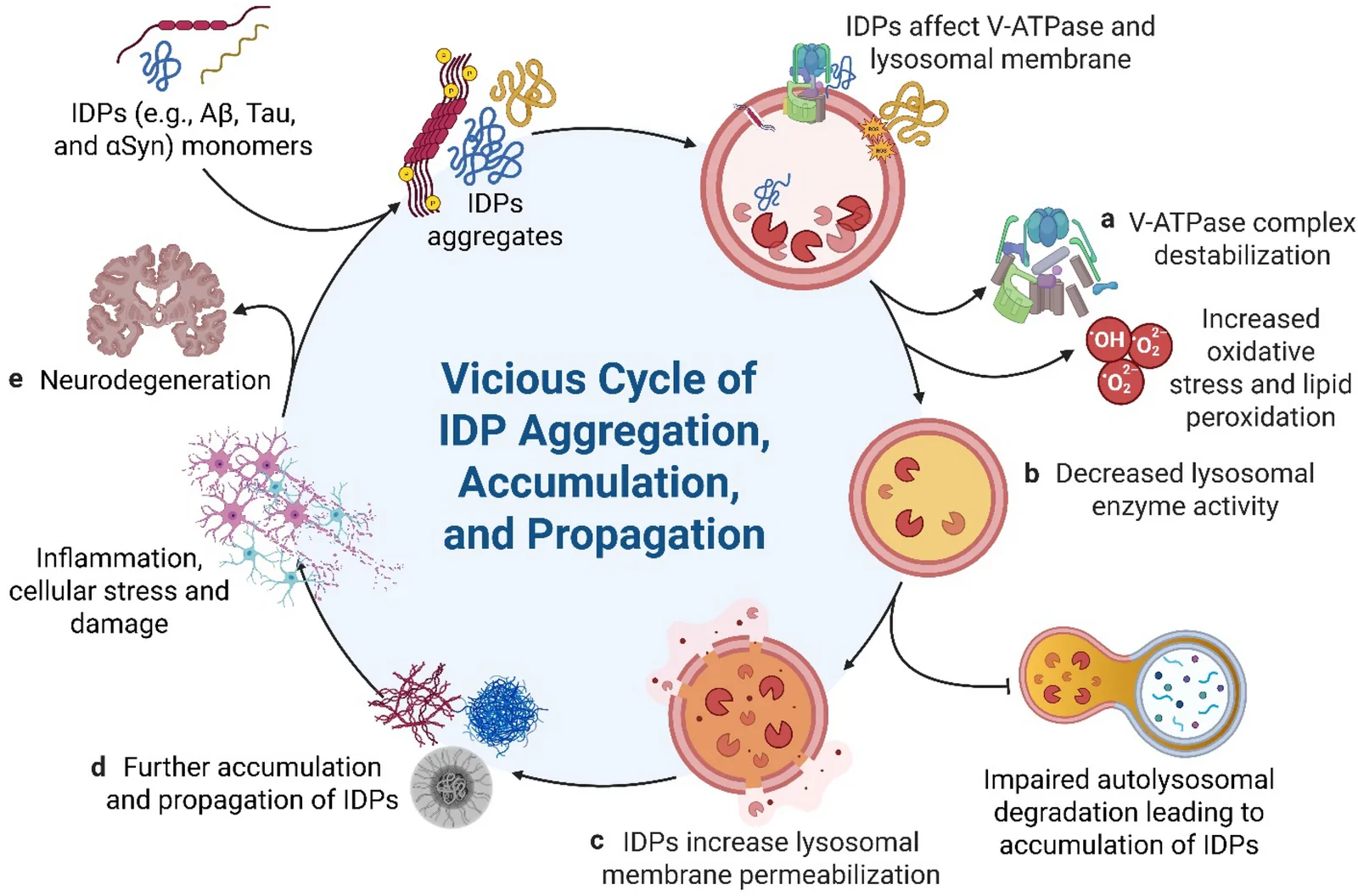
Schematic representation of the interplay between IDP aggregation and lysosomal dysfunction in neurodegeneration. **a** IDPs disrupt lysosomal V-ATPase function, leading to impaired proton pumping and lysosomal deacidification, while also promoting oxidative stress and lipid peroxidation that can further compromise lysosomal function. **b** These changes reduce lysosomal hydrolase activity and overall degradative capacity. **c** Impaired lysosomal degradation compromises autophagic clearance, resulting in the accumulation of undegraded cargo and pathogenic IDPs and increasing susceptibility to lysosomal membrane permeabilization. **d** Lysosomal membrane permeabilization causes leakage of luminal contents and further disrupts lysosomal homeostasis, thereby exacerbating proteostatic stress and IDP accumulation. **e** These defects promote inflammation, cellular stress, and neuronal damage, ultimately contributing to progressive neurodegeneration. Created with Biorender.com

The accumulation of these aggregates disrupts cellular homeostasis, impairs intracellular trafficking and synaptic function, and ultimately contributes to neuronal dysfunction and death [32–35]. In particular, several IDPs, including Aβ, tau, and αSyn, have been shown to disrupt lysosomal membrane integrity and function, further compromising the degradation of misfolded proteins [38–40]. Impaired lysosomal degradation, in turn, promotes the accumulation of these aggregation-prone proteins [41, 42], contributing to a vicious cycle of IDP aggregation, accumulation, and propagation that ultimately drives neurodegeneration (Fig. 1). The intrinsically disordered nature of IDPs also enables them to undergo liquid–liquid phase separation, forming membrane-less condensates that concentrate specific proteins and biomolecules [43–45]. While liquid–liquid phase separation plays important roles in cellular organization and regulation, pathological conditions can drive aberrant phase transitions in which IDPs, including Aβ, tau, and αSyn, form condensates that may mature into less dynamic, aggregation-prone states. These transitions are increasingly implicated in the formation of insoluble inclusions and the disruption of lysosomal and autophagic function observed in neurodegenerative diseases [45–47].

These biophysical properties of IDPs, including their propensity for aggregation and phase separation, contribute to the mechanisms by which they impair lysosomal function. Aggregated species, including oligomeric and fibrillar forms, have been shown to interfere with V-ATPase function, destabilizing the complex and impairing proton translocation [7, 48] (Fig. 1a). In parallel, these aggregates induce oxidative stress and lipid peroxidation, which further compromise lysosomal enzyme activity (Fig. 1a). In sum, these effects reduce lysosomal acidification and degradative capacity (Fig. 1b). In addition, several IDPs promote lysosomal membrane permeabilization (LMP) (Fig. 1c), resulting in leakage of lysosomal enzymes and further accumulation of undegraded substrates [38, 39, 49–53] (Fig. 1d). These disruptions contribute to proteostasis imbalance, inflammatory signaling, and cellular dysfunction or death [54, 55] (Fig. 1e). Consistent with this, lysosomal impairment is increasingly recognized as a central contributor to progressive neuronal loss [41, 42, 56, 57]. Similar defects are also observed in glial cells, including astrocytes and microglia, where impaired lysosomal function may further limit the clearance and containment of toxic protein aggregates [58, 59].

Accumulation of IDPs such as Aβ, tau, and αSyn further amplifies pathology through impaired clearance and intercellular propagation. When lysosomal degradation is compromised, aggregates can be released via soluble N-ethylmaleimide-sensitive factor attachment protein receptor (SNARE)-dependent, Ca^2+^-regulated lysosomal exocytosis. Once in the extracellular space, these species exhibit prion-like behavior, seeding aggregation and promoting neuron-to-neuron and neuron-to-glia spread [60–62]. Glial cells also contribute to this process by internalizing extracellular aggregates, which can trigger proinflammatory cytokine release and, under conditions of lysosomal dysfunction, further enhance extracellular aggregate accumulation [63]. Thus, lysosomal exocytosis represents a key link between intracellular proteostasis failure and the spread of pathology across neural networks.

Beyond Aβ, tau, and αSyn, other IDPs implicated in neurodegeneration are also associated with lysosomal dysfunction. Huntingtin, the pathogenic protein in Huntington’s disease, interacts with V-ATPase subunits in the striatum of mouse models, disrupting lysosomal acidification and autophagic function [64]. Similarly, TDP-43, a major pathological protein in amyotrophic lateral sclerosis and FTD, impairs autophagosome-lysosome fusion, thereby promoting its own accumulation [65]. These findings suggest that IDP-driven lysosomal dysfunction represents a shared feature across multiple neurodegenerative diseases.

In this review, we focus on Aβ, tau, and αSyn, as these proteins have the most well-established links to V-ATPase-dependent lysosomal acidification defects across in vitro, in vivo, and human studies, and underlie the most prevalent neurodegenerative disorders worldwide [66]. We examine how these IDPs disrupt V-ATPase integrity and function within the broader lysosomal system, impair acidification, and compromise autophagy-lysosomal pathway dynamics. We further discuss how these defects propagate through interconnected processes, including altered proteostasis, lysosomal membrane instability, and impaired degradation, ultimately contributing to neuronal and glial dysfunction, inflammation, and disease progression. Finally, we highlight emerging therapeutic strategies aimed at restoring lysosomal acidification and function, enhancing V-ATPase function, and mitigating IDP-driven neurotoxicity.

## Aβ-induced lysosomal dysfunction and autophagic blockade

**Fig. 2:**
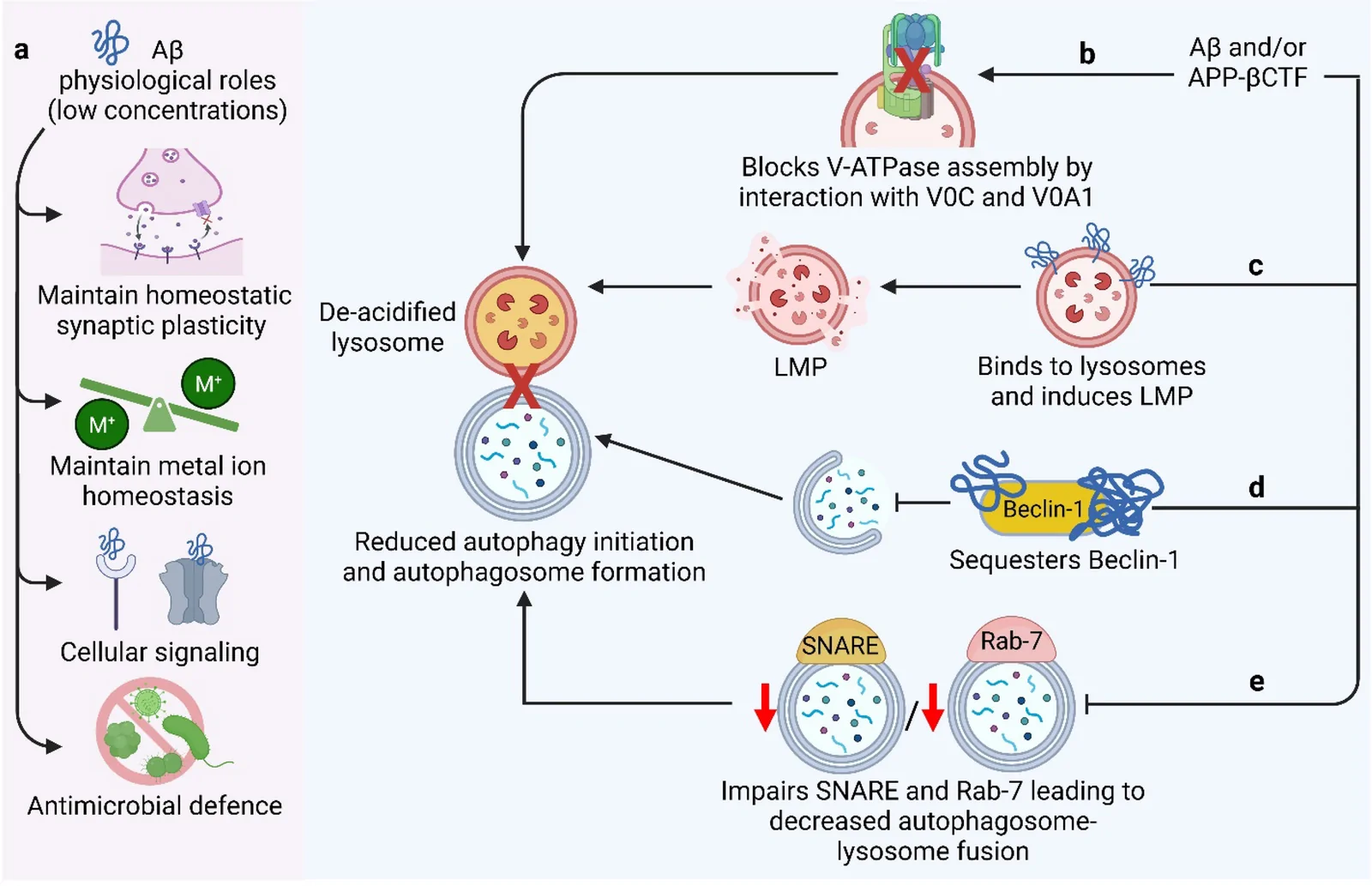
Schematic representation of the dual roles of Aβ in physiological and pathological processes. **a** At physiological levels, Aβ has been implicated in several cellular functions, including the regulation of synaptic plasticity, metal ion homeostasis, cellular signaling, and antimicrobial defense. **b** Under pathological conditions, Aβ and APP-βCTF impair lysosomal acidification, including through interactions with V-ATPase subunits such as ATP6V0C and ATP6V0A1, and may interfere with V-ATPase assembly and function. **c** Aβ can also associate with lysosomal membranes, promoting lysosomal membrane permeabilization (LMP) and further disrupting lysosomal integrity and acidification. **d** Aβ can sequester Beclin-1, reducing its availability for autophagy initiation and thereby impairing autophagosome formation. **e** Aβ further disrupts autophagosome-lysosome fusion by interfering with components of the vesicle trafficking and membrane fusion machinery, including SNARE proteins and Rab7. Created with Biorender.com

Aβ has been implicated in several physiological processes at low concentrations, including cell adhesion, synaptic plasticity, metal ion homeostasis, cellular signaling, and antimicrobial defense [67–69] (Fig. 2a). The stability of Aβ fibrils is primarily driven by intermolecular hydrogen bonding and steric zipper interactions between β-sheets [70]. Structural heterogeneity, often referred to as amyloid polymorphism, arises from differences in β-strand orientation, packing, and protofilament organization, and is thought to influence aggregation kinetics, toxicity, and disease progression in neurodegenerative disorders [71]. Pathogenic Aβ species play a central role in neurodegeneration. Aβ exists as multiple isoforms, including Aβ_40_ and the more aggregation-prone Aβ_42_, and can adopt conformations that differ in stability and pathogenic potential [72–77]. Disease-associated variants may arise from mutations such as the Arctic E22G substitution in Aβ and the London V717I mutation in APP [78], as well as from truncations [79, 80], or post-translational modifications, including pyroglutamate formation [81], phosphorylation [82], and oxidation. These alterations generally enhance the aggregation propensity, increase toxicity, and reduce susceptibility to proteolytic clearance, thereby contributing to disease progression and highlighting pathological Aβ species as important targets for diagnostic and therapeutic strategies in AD [83, 84].

### Aβ and APP-βCTF as mediators of faulty lysosomal acidification

Aβ_42_ is a pathogenic peptide that plays a central role in AD. It is generated through amyloidogenic processing of APP, in which APP is sequentially cleaved by β-secretase and γ-secretase. The β-secretase cleavage releases soluble APPβ and the membrane-bound β-C-terminal fragment (β-CTF, also called C99). This fragment is then processed by γ-secretase through stepwise intramembrane cleavage, producing the APP intracellular domain and Aβ peptides of varying lengths, most commonly Aβ_40_ and the more aggregation-prone Aβ_42_ [85, 86]. Owing to its higher hydrophobicity and greater tendency to self-associate, Aβ_42_ is considered particularly pathogenic and contributes to extracellular amyloid plaque formation, a defining feature of AD. Accumulation of Aβ, driven by an imbalance between production and clearance, is a key factor in disease progression [85, 86]. Increasing evidence indicates that impaired protein clearance, especially lysosomal dysfunction, plays a critical role in this process [42, 87].

Aβ and APP-βCTF interfere with V-ATPase activity through interactions on distinct sides of the lysosomal membrane (Fig. 2b). Aβ has been reported to bind V-ATPase subunits such as ATP6V0C via intraluminal regions (Δ82–85), involving residues D82, D83, I84, and S85, suggesting luminal-side interactions that impair V-ATPase function [7]. Aβ is also found in ATP6V0C-positive vesicles in hippocampal neurons in human brains, and these vesicles are enlarged in AD compared to controls, supporting a potential functional association [7]. Phosphorylated APP-βCTF (pY682-APP-βCTF) directly binds a defined pocket within the cytoplasmic domain of the ATP6V0A1 subunit (involving residues F196 and R167) [10]. This interaction is mediated by the YENPTY motif, particularly phosphorylated Tyr682, and interferes with V1-V0 association [10]. As a result, V-ATPase assembly is impaired, leading to reduced ATP hydrolysis and decreased proton translocation into lysosomes. Reducing APP-βCTF levels or its phosphorylation restores V-ATPase activity and lysosomal acidification, highlighting a potential therapeutic avenue [10]. In sum, these findings suggest complementary mechanisms: APP-βCTF acts on the cytosolic side via ATP6V0A1, whereas Aβ acts from the luminal side, likely via ATP6V0C. In addition, Aβ can insert into lysosomal membranes, promoting LMP and proton leakage, thereby increasing luminal pH [49] (Fig. 2c). The resulting loss of proton and ion gradients across the lysosomal membrane impairs acidification, which in turn compromises lysosomal enzyme activities [88], leading to inefficient degradation of autophagic cargo. As a result, undegraded substrates, including Aβ and damaged organelles, accumulate and further exacerbate cellular dysfunction [42].

### Pathways of Aβ- and APP-βCTF-induced lysosomal and autophagy impairments

Aβ reduces nuclear translocation of TFEB, a master regulator of lysosomal biogenesis, and decreases expression of downstream targets such as OSTM1 (osteoporosis-associated transmembrane protein 1), thereby compromising lysosomal function in primary microglial cells [89]. Aβ_42_ oligomers also disrupt the endosomal-lysosomal organization, leading to mislocalization of Rab7- and LAMP1-positive vesicles and Golgi alterations. This vesicular disorganization impairs trafficking and delays degradation of internalized cargo [90]. Amyloid-like β-sheet aggregates (β4 and β23), which model aggregation-prone proteins such as Aβ, impair late-stage autophagy by sequestering AP-3μ1, disrupting AP-3-dependent lysosomal trafficking and causing cargo missorting [91]. In aged and AD transdifferentiated neurons, Aβ accumulation is linked to impairment of ESCRT-mediated lysosomal membrane repair, leading to persistent lysosomal damage, proteostasis failure, inflammatory signaling, and cell death [92].

Additional evidence points to impaired autophagic flux in Aβ pathology. Aβ oligomers disrupt mTORC1 signaling, affecting proper regulation of autophagy [93, 94]. Aβ can also sequester Beclin-1, a key regulator of autophagosome nucleation, reducing its availability for autophagosome nucleation [94] (Fig. 2d). Furthermore, Aβ interferes with autophagosome-lysosome fusion by disrupting key trafficking and fusion proteins, including SNAREs and Rab GTPases (Fig. 2e). In particular, Aβ impairs Rab7, a small GTPase required for late-stage autophagy, through mislocalization or functional inactivation [95]. These changes impair lysosomal clearance and promote Aβ accumulation, while accumulated Aβ further compromises lysosomal and autophagic function [41, 42]. Consistent with this, several AD mouse models show impaired lysosomal acidification. The Ts2 Down syndrome model, characterized by *APP* gene triplication and elevated β-CTF levels [10], exhibits fewer fully acidified autolysosomes. APP^NL−G−F^ mice display reduced expression of lysosomal V-ATPase subunits [96], and multiple AD models exhibit reduced lysosomal acidification in neurons [42]. Reactive oxygen species further contribute by inducing lipid peroxidation of lysosomal membranes, destabilizing conditions required for optimal V-ATPase function [97]. In human AD CA1 pyramidal neurons, accumulation of LC3-II and SQSTM1/p62, along with enlarged autolysosomes, is consistent with impaired autophagic clearance [98].

Genetic factors also modulate these processes. Mutations in presenilin-1 (*PSEN1*), linked to familial AD, have been reported to impair V-ATPase trafficking and lysosomal acidification while increasing Aβ_42_ production [99]. Early work proposed that PSEN1 acts as an ER chaperone for the V0a1 subunit, with loss leading to defective lysosomal targeting and reduced V-ATPase activity [23]. However, subsequent studies using presenilin-deficient models suggest that lysosomal pH and cathepsin D maturation are largely preserved, arguing against a primary defect in acidification [100]. Instead, PSEN1 loss induces compensatory upregulation of CLEAR network genes under TFEB control, including *LAMP1*, *CTSD*, and *ATP6V0D1*, consistent with adaptive lysosomal remodeling [100, 101]. *PSEN1* mutations have also been linked to altered lysosomal Ca^2+^ release [102], which may intersect with pH-dependent regulation of lysosomal function [103]. Overall, these findings support a more nuanced role for PSEN1 in lysosomal homeostasis.

Which Aβ species, monomers, oligomers, or fibrils, drive lysosomal dysfunction remains unresolved. Current evidence points to oligomers as the primary toxic species. Aβ_42_ preferentially forms oligomers that associate with membranes, whereas Aβ_40_ more often forms lower-order assemblies such as dimers [49]. The toxicity of these interactions is supported by observations that membrane insertion of oligomeric Aβ_42_ correlates with increased cellular toxicity, while the less structured Aβ_40_ exhibits reduced membrane-disruptive capacity [104]. Although Aβ has been reported to interact with V-ATPase subunits such as ATP6V0C, the specific species responsible and the functional consequences remain unclear. Importantly, it is also not established whether the interaction is mediated directly by intrinsically disordered regions (IDRs) of Aβ or instead results from conformational changes associated with oligomerization, membrane binding, or other structural states. Thus, while the dynamic and conformationally heterogeneous nature of Aβ may facilitate transient interactions with lysosomal proteins, direct IDR-mediated binding to lysosomal targets has not been demonstrated. Beyond lysosomal effects, Aβ oligomers impair autophagy more broadly by disrupting axonal transport of autophagosomes, contributing to cellular stress and autophagic failure [105]. Although both monomeric and oligomeric Aβ can affect autophagy, oligomers are more consistently associated with cytotoxic outcomes, including necroptosis and apoptosis [49]. In contrast, fibril-associated toxicity may relate more strongly to resistance to degradation than to direct interference with lysosomal machinery. In sum, current evidence supports a prominent role for oligomeric Aβ in lysosomal and autophagic dysfunction, but the species-specific mechanisms and, importantly, the contribution of Aβ IDRs to direct interactions with lysosomal acidification machinery remain to be defined.

## Tau-driven autophagy and lysosomal dysfunction

Tau is a microtubule-associated protein encoded by the *MAPT* gene that plays a central role in stabilizing microtubules, regulating cytoskeletal dynamics, supporting intracellular transport and processes essential for neuronal plasticity and development [106–108]. In the adult human brain, alternative splicing of *MAPT* gives rise to six tau isoforms that differ in the number of microtubule-binding repeats and N-terminal inserts [109]. Pathogenic mutations in *MAPT*, such as P301L, P301S, and R406W, are linked to FTD, including FTDP-17 (frontotemporal dementia with parkinsonism linked to chromosome 17), highlighting the role of tau dysfunction in neurodegeneration [110, 111]. Tau undergoes a range of abnormal post-translational modifications (PTMs) that promote its dissociation from microtubules and initiate aggregation into soluble and insoluble species, a hallmark of tauopathies. Among these, hyperphosphorylation is one of the most extensively characterized. Phosphorylation at residues such as Ser202 and Thr231 reduces tau’s affinity for microtubules, leading to cytoskeletal destabilization and facilitating aggregation into oligomers and fibrils [112, 113]. Proteolytic truncation, particularly at sites such as Glu391 and Asp421, generates aggregation-prone fragments with increased neurotoxicity [114, 115]. Additional PTMs, including acetylation, glycation, and ubiquitination, further modulate tau stability, aggregation propensity, and toxicity [116, 117].

### Disruption of autophagy-lysosomal pathways by tau aggregation

**Fig. 3:**
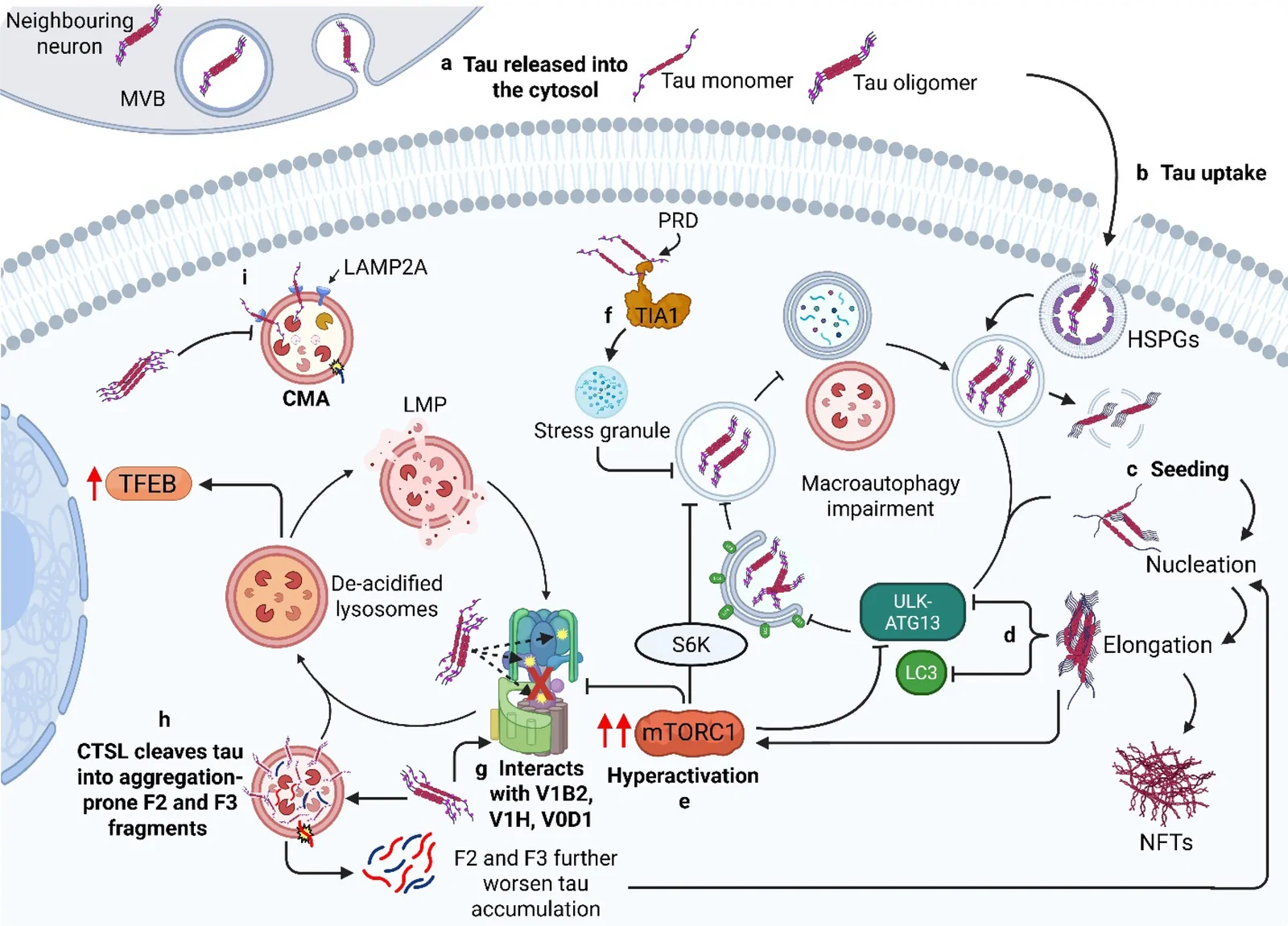
Schematic overview of tau pathology and autophagy-lysosomal dysfunction. **a, b** Pathological tau can be released from neurons through regulated secretion and extracellular vesicle-associated pathways (**a**) and internalized by neighboring cells, in part through interactions with cell-surface heparan sulfate proteoglycans (HSPGs) (**b**). **c** Following uptake, extracellular tau seeds the conformational conversion and aggregation of endogenous tau, promoting the formation of oligomeric and fibrillar species and, ultimately, neurofibrillary tangles (NFTs). **d** Tau accumulation impairs macroautophagy, including disruption of the ULK1-ATG13 complex and altered LC3-II levels, thereby compromising autophagy initiation and flux. **e** Tau also promotes mTORC1 hyperactivation and downstream S6K signaling, further suppressing autophagy and exacerbates tau accumulation. **f** Tau interacts with the prion-like, poly-glycine-rich domain (PRD) of TIA1, promoting stress-granule formation and altering proteostasis pathways, which may further impair autophagic clearance. **g** Pathological tau interacts with V-ATPase subunits, including ATP6V1B2, ATP6V1H, and ATP6V0D1, and impairs V-ATPase function, resulting in reduced lysosomal acidification. **h** Tau-associated lysosomal dysfunction can compromise lysosomal proteolytic activity and membrane integrity, facilitating cathepsin L (CTSL)-mediated cleavage of tau into aggregation-prone F2 and F3 fragments. **i** Tau also interacts with LAMP2A at the lysosomal membrane and can interfere with chaperone-mediated autophagy (CMA), thereby further limiting tau degradation and clearance. Created with Biorender.com

Under physiological conditions, tau is predominantly localized to axons. In disease states, it redistributes to somatodendritic and other compartments, where it disrupts signaling pathways and cellular homeostasis. This mislocalization occurs early and precedes NFT formation [118]. Tau aggregation and spread are thought to involve templated misfolding through nucleation and elongation, analogous to prion-like propagation [38, 119–121]. Initially, misfolded tau forms oligomers and subsequently paired helical filaments, with larger assemblies often showing greater seeding capacity. During elongation, these seeds recruit soluble tau, promoting conformational conversion and incorporation into growing aggregates, ultimately leading to NFTs [38, 119–121]. Tau aggregates can be released into the extracellular space and taken up by neighboring cells, where they template further aggregation of endogenous tau (Fig. 3a). This propagation is mediated in part by tau binding to heparan sulfate proteoglycans, facilitating uptake via macropinocytosis [122–124] (Fig. 3b). Once internalized, tau seeds drive nucleation, aggregation, and fibrillization of endogenous tau (Fig. 3c).

The accumulation and spread of tau aggregates are closely tied to autophagic-lysosomal clearance. Tau is degraded via macroautophagy, where aggregated tau is delivered to lysosomes via autophagosomes, and chaperone-mediated autophagy (CMA), which targets monomeric tau through LAMP2A [125]. Tau overexpression has been reported to impair autophagy initiation and flux, with reduced LC3-II levels, disrupted ULK1-ATG13 interactions (Fig. 3d), and mTORC1 hyperactivation observed in HEK293T and N2A cells (Fig. 3e). This hyperactivation, associated with phosphorylation of 4EBP1, p70S6K1, and ULK1, along with elevated intracellular amino acid levels, suppresses autophagy and promotes tau accumulation [6]. Enhanced mTORC1-S6K signaling further suppresses autophagic activity, thereby exacerbating tau pathology [6]. Tau also perturbs proteostasis through interactions with TIA1 (T-cell intracellular antigen 1) (Fig. 3f), promoting stress granule formation and sequestering RNA-binding proteins, which may indirectly compete with autophagy for cellular resources [126]. Glial cells can take up extracellular tau and may contribute to its spread across cell types [127, 128]. Defects in autophagy, lysosomal function, and trafficking impair tau clearance and promote its accumulation, further contributing to tau propagation and neurodegeneration.

### Tau-induced dysregulation of V-ATPase activity and defective lysosomal acidification

Tau appears to disrupt lysosomal acidification through interactions with V-ATPase subunits on both cytosolic and luminal sides of the lysosomal membrane [7, 129]. In primary hippocampal neurons, it was shown that phosphorylated tau (p-tau), especially the PHF-1-reactive form phosphorylated at S396/S404, interacts with the cytosolic V-ATPase subunit ATP6V1B2. Mapping experiments showed that deleting the N-terminal, nucleotide-binding, or C-terminal regions of ATP6V1B2 did not disrupt this interaction, whereas deletion of residues 120–172 abolished tau binding. This places the tau-interaction site within ATP6V1B2 residues 120–172, a region adjacent to the ATP-binding domain. This pathogenic interaction reduces V-ATPase activity and impairs lysosomal acidification [7]. Interactome studies using AD brain tissue further showed associations between p-tau and V-ATPase subunits ATP6V1B2 and ATP6V1H (V1 domain), as well as ATP6V0D1 (V0 domain), suggesting that p-tau may interfere with V-ATPase function at multiple sites [129] (Fig. 3g). In iPSC-derived neurons expressing tau mutations (*P301L*, *P301S*, *R406W*), reduced expression of V-ATPase subunits and increased lysosomal pH have been reported [130–133]. Similar reductions in V-ATPase subunits (e.g., ATP6V1E1) are observed in tau transgenic mouse models [134, 135].

Beyond direct V-ATPase interactions, tau can indirectly impair lysosomal acidification by compromising lysosomal membrane integrity. Tau insertion into lysosomal membranes induces nanoscale damage and LMP, leading to leakage of protons and hydrolases into the cytosol and dissipation of the proton gradient [38]. Release of lysosomal cathepsins can further activate both caspase-dependent and independent apoptotic pathways [136, 137]. Tau can also enter lysosomes via CMA. During this process, its C-terminus is translocated through LAMP2A, where it can be cleaved by cathepsin L to generate aggregation-prone fragments (F2 and F3) that remain associated with the lysosomal membrane (Fig. 3h). These partially translocated species can oligomerize at the membrane interface and interfere with CMA substrate uptake, further impairing lysosomal function [138] (Fig. 3i). Other truncated tau species, such as tau35, inhibit TFEB nuclear translocation, reducing lysosomal biogenesis [139]. Mutant tau also disrupts CMA by interacting with LAMP2A and impairing translocation [138]. Acetylated tau, while still able to bind the lysosomal membrane, shows reduced interaction with Hsc70, decreasing delivery efficiency and degradation [140].

Tau clearance also involves ESCRT-dependent endolysosomal pathways [141]. Tau can be redirected from macroautophagy to multivesicular bodies (MVBs), where degradation depends on late ESCRT components such as ESCRT-III [141]. Disruption of ESCRT proteins, particularly CHMP6, compromises membrane integrity and allows tau seeds to escape into the cytosol, promoting prion-like propagation [142]. The BAG3-Rab35-ESCRT axis plays a regulatory role in this process. BAG3, together with HSP70, maintains Rab35 activity by inhibiting its GAP TBC1D10B, thereby promoting ESCRT recruitment and facilitating tau sorting into endosomes and MVBs for lysosomal degradation [143]. Disruption of this pathway reduces tau clearance, whereas BAG3 upregulation enhances association of phosphorylated tau with ESCRT components (e.g., CHMP2B) and lowers tau burden [143]. This pathway exhibits selectivity for specific phospho-tau species, preferentially targeting pSer396/404 and pSer262 over pSer202 [144]. ESCRT function also contributes to autophagic flux. Tau accumulation suppresses IST1 expression, impairing ESCRT-III assembly and autophagosome-lysosome fusion. Mechanistically, tau represses IST1 transcription via ANP32A-dependent chromatin effects, further compromising lysosomal degradation [145].

Tau toxicity depends on its conformational and post-translational state, including monomeric, oligomeric, and fibrillar forms. While monomeric tau may interact with V-ATPase, current evidence more consistently implicates phosphorylated tau in these interactions [7, 129]. Phosphorylation can promote tau misfolding and aggregation, increasing the likelihood of higher-order species [146]; however, the conformational state of tau responsible for interaction with V-ATPase has not been clearly established. Likewise, it remains unclear whether these interactions are mediated directly by tau IDRs or by conformational surfaces that emerge upon phosphorylation, misfolding, or oligomerization. Tau fibrils accumulating within lysosomes appear to exert toxicity primarily through nanoscale membrane damage, leading to proton leakage, lysosomal alkalinization, and eventual escape of tau into the cytosol [38]. Recent work suggests that this membrane damage may involve transient, reversible nanoscale pores rather than catastrophic membrane rupture [50]. Thus, distinct tau species may affect lysosomal function through different mechanisms, with phosphorylated and potentially higher-order tau species implicated in V-ATPase dysfunction, whereas fibrillar tau appears more closely associated with lysosomal membrane damage and cytosolic seeding. However, because many studies do not directly resolve the conformational state of the interacting tau species, these distinctions should be interpreted cautiously.

## αSyn-mediated autophagic failure, lysosomal and V-ATPase dysfunction

αSyn is a 140-amino-acid protein composed of three functionally distinct domains: an N-terminal amphipathic region, a central non-amyloid-β component (NAC) domain, and an acidic C-terminal domain [147]. In solution, monomeric αSyn is largely unfolded and is therefore classified as an IDP [148]. However, its structural ensemble remains debated, with conflicting reports in the literature. Environmental conditions, disease-associated mutations, and interactions with lipids or proteins can shift αSyn toward distinct conformations, reflecting its structural plasticity. Misfolding and aggregation into β-sheet-rich amyloid fibrils are central features of synucleinopathies, including PD [148]. Two models have been proposed for the native state of αSyn: a predominantly unstructured monomer [149], and a metastable, α-helical tetramer [150]. Most subsequent work supports the monomeric model, in which αSyn exists mainly as a random coil in the cytosol and adopts α-helical structure upon membrane binding [151]. Nevertheless, its precise physiological conformation remains unresolved.

Functionally, αSyn regulates synaptic vesicle dynamics, including neurotransmitter storage, recycling, and release [152, 153]. It has also been linked to modulation of enzymatic activity and may influence dopamine transporter function [154]. αSyn associates with cellular membranes, including synaptic vesicles and mitochondria-associated membranes, an ER subdomain involved in lipid and Ca^2+^ homeostasis [40, 155]. Upon membrane binding, αSyn adopts an α-helical conformation that facilitates interactions with vesicular lipids and proteins such as VAMP2 (vesicle-associated membrane protein 2) and other SNARE components [156]. Through these interactions, αSyn contributes to synaptic vesicle trafficking, neurotransmission, neuronal plasticity, and mitochondrial function, although these processes become disrupted under pathological conditions [157].

Pathological aggregation of αSyn arises from genetic, biochemical, and environmental factors. Familial *SNCA* mutations (A30P, E46K, H50Q, A53T), along with post-translational modifications such as phosphorylation at Ser129, increase aggregation propensity [158–160]. Additional modifications, including nitration, glycation, and ubiquitination, further reduce solubility and impair clearance [161–163]. Under these conditions, αSyn transitions from monomeric or membrane-bound α-helical states to β-sheet-rich oligomeric and fibrillar assemblies. These aggregated species disrupt synaptic vesicle recycling, impair neurotransmission, particularly dopaminergic signaling, and contribute to dendritic spine loss. Even in the absence of mutations, αSyn has an intrinsic tendency to aggregate, largely due to the hydrophobic NAC domain [164]. Efficient clearance of misfolded αSyn is therefore essential for neuronal proteostasis. Consistent with this, lysosomal dysfunction is a major contributor to αSyn accumulation. Loss-of-function mutations in lysosomal genes such as *GBA1* [165] and *TMEM175* [166], are linked to increased αSyn aggregation and neurotoxicity. TMEM175, a lysosomal potassium channel, helps maintain ionic conditions required for V-ATPase activity and lysosomal acidification. Its impairment disrupts lysosomal pH maintenance, reduces degradative capacity, and promotes αSyn accumulation, thereby exacerbating lysosomal dysfunction [12]. Thus, αSyn aggregation both results from and contributes to lysosomal dysfunction.

### Destabilization of lysosomal membranes and disruption of enzyme trafficking by αSyn aggregates

**Fig. 4:**
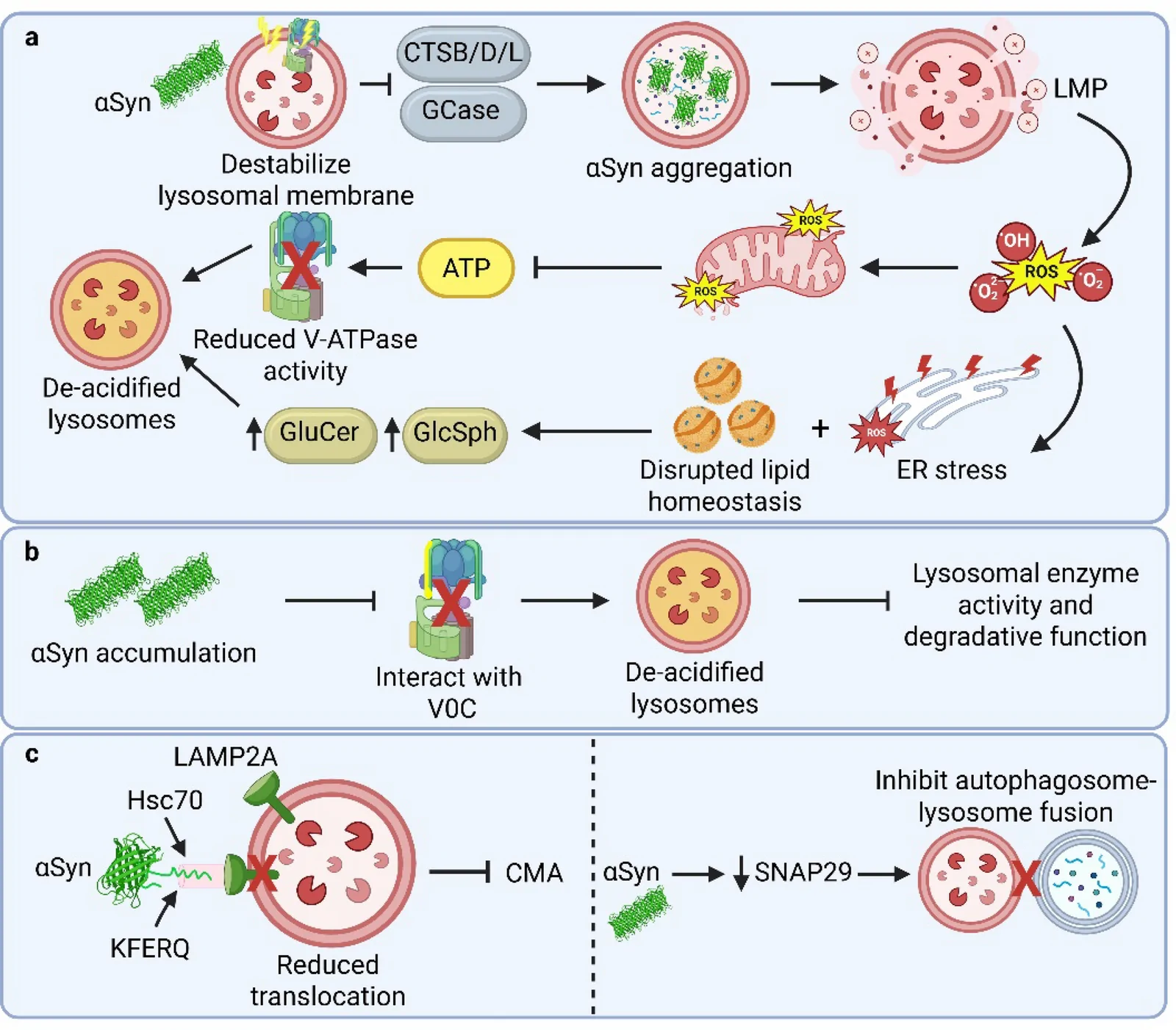
Schematic representation of αSyn-mediated lysosomal and autophagy dysfunction. **a** Aggregated αSyn destabilizes endolysosomal membranes, promoting lysosomal membrane permeabilization (LMP) and facilitating αSyn seeding and propagation. LMP can result in leakage of protons and lysosomal proteases into the cytosol, disrupting lysosomal homeostasis and contributing to oxidative stress, mitochondrial dysfunction, and ER stress, thereby further increasing cellular stress. In parallel, αSyn impairs lysosomal enzyme trafficking and activity, reducing the function of key hydrolases, including cathepsins (CTSB/D/L) and glucocerebrosidase (GCase). Altered lysosomal lipid homeostasis, including accumulation of glucosylceramide (GlcCer) and related sphingolipids (e.g., glucosylsphingosine (GlcSph)), can further compromise lysosomal function. **b** αSyn disrupts lysosomal acidification by interfering with the V-ATPase complex. Pathogenic αSyn species interact with V-ATPase subunits, including ATP6V0C, and impair V-ATPase assembly and/or function, leading to reduced proton pumping, impaired autophagic flux, and decreased lysosomal degradative capacity. **c** αSyn impairs both chaperone-mediated autophagy (CMA) (left) and macroautophagy (right). Misfolded αSyn is recognized by Hsc70 through a KFERQ-like motif and binds the lysosomal receptor LAMP2A; under pathological conditions, however, αSyn can accumulate at the lysosomal membrane and interfere with its translocation, thereby impairing CMA. Concurrently, αSyn disrupts SNARE-dependent autophagosome-lysosome fusion, including through reduction of SNAP29, resulting in autophagosome accumulation and impaired autolysosome formation. Created with BioRender.com

Accumulating evidence indicates that pathogenic αSyn species directly compromise lysosomal membrane integrity and degradative capacity, promoting LMP and enhancing αSyn seeding [51] (Fig. 4a). Exogenous αSyn can enter cells via direct membrane penetration or endocytosis, after which endo-lysosomal rupture enables cytosolic aggregation. αSyn aggregates from diffuse Lewy body disease (DLBD) brains preferentially induce endocytosis-dependent seeding coupled to vesicle rupture and show lower seeding potency than recombinant aggregates. Co-localization of αSyn pathology with galectin-3, a marker of vesicle rupture, in DLBD brain tissue supports a role for endo-lysosomal damage in human disease [51]. Cells exhibiting LMP are more susceptible to αSyn seeding, suggesting that membrane damage facilitates disease progression. This membrane-disruptive effect is supported by studies showing that αSyn fibrils induce relocalization of galectin-3 from diffuse cytosol to punctate vesicular structures, indicative of vesicle rupture, whereas monomeric αSyn does not [52]. Importantly, vesicle rupture appears to be a shared property of pathogenic αSyn assemblies, including familial mutants and distinct conformational strains, and is not unique to αSyn, as similar effects are observed with tau and huntingtin aggregates [53]. αSyn also perturbs phosphoinositide metabolism. It interacts with negatively charged lipids such as PIP2 and PIP3, adopting a helical conformation and redistributing in response to PI3K (phosphoinositide 3-kinase) signaling [167]. Lysosomal membranes are enriched in PI(3,5)P_2_, a lipid that regulates organelle morphology and acidification [168]. Interference with this lipid environment, for example, through altered PI(3,5)P_2_ levels, can disrupt lysosomal function. Inhibition of PIKfyve alters PI(3,5)P_2_ and can induce lysosomal hyperacidification, which itself can impair lysosomal function [169].

In addition to membrane effects, αSyn impairs lysosomal enzyme trafficking. In patient-derived neurons and mouse models, αSyn aggregation disrupts delivery and maturation of cathepsins (CTSB, CTSD, CTSL), reducing proteolytic activity. Enhancing hydrolase trafficking via the SNARE protein ykt6 restores cathepsin maturation and lowers αSyn levels [170]. Membrane-associated αSyn can also directly interact with glucocerebrosidase (GCase), inhibiting its activity [171]. These defects reduce lysosomal degradative capacity, leading to accumulation of undegraded substrates [172]. αSyn-induced LMP further causes leakage of lysosomal enzymes into the cytosol, triggering stress responses. This includes increased reactive oxygen species, contributing to oxidative stress and mitochondrial dysfunction [157] (Fig. 4a). Reduced mitochondrial ATP production limits energy available for V-ATPase activity, further impairing lysosomal acidification [58, 59]. In parallel, αSyn disrupts the ER-mitochondria coupling, altering Ca^2+^ homeostasis and contributing to cellular stress [173]. Mutations in *GBA1* exacerbate these effects by altering glycosphingolipid metabolism and promoting accumulation of glucosylceramide and glucosylsphingosine, which further destabilize lysosomal function [174–176].

### αSyn-induced autolysosomal dysfunction and repair deficits

αSyn aggregation disrupts lysosomal acidification, autophagic flux, and membrane repair. A central mechanism involves V-ATPase dysfunction. αSyn preformed fibrils interact with ATP6V0C, interfering with V0-V1 assembly and reducing ATP6V0C expression. This leads to defective acidification, impaired autophagic flux, and reduced αSyn degradation in microglia [48] (Fig. 4b). Restoration of ATP6V0C rescues lysosomal acidity and enhances αSyn clearance [48]. Loss of ATP6V0C similarly promotes αSyn accumulation and cellular toxicity [20]. Exosomes from PD patients enriched in oligomeric αSyn downregulate ATP6V1G1 in microglia, causing lysosomal enlargement, autophagosome accumulation, and intracellular αSyn buildup [177]. In cellular models, αSyn accumulates within the endolysosomal system and alters lysosomal morphology. While αSyn co-localizes with endosomal and lysosomal markers regardless of aggregation state, oligomeric and fibrillar forms induce enlarged lysosomes and reduced cathepsin D activity, consistent with impaired function [178]. In dopaminergic neurons, wild-type αSyn reduces long-lived proteolysis in an aggregation-dependent manner, whereas aggregation-deficient mutants have minimal effect. Activities of multiple lysosomal enzymes, including CTSB, GCase, β-galactosidase, and hexosaminidase, are also reduced [179].

Beyond acidification defects, αSyn interferes with core autophagic pathways (Fig. 4c). It is degraded via macroautophagy [180, 181] and CMA [182]. Macroautophagy clears aggregated αSyn through autophagosome formation and lysosomal fusion [180, 181], whereas CMA selectively degrades soluble αSyn via a KFERQ-like motif recognized by Hsc70 and delivered to LAMP2A [183]. Under pathological conditions, misfolded αSyn binds LAMP2A with high affinity but fails to translocate, leading to CMA blockade [184]. αSyn also disrupts autophagosome-lysosome fusion by reducing SNAP29 levels, leading to autophagosome accumulation [185]. In addition, αSyn perturbs mTORC1 signaling by interacting with TSC2 and destabilizing the TSC1-TSC2 complex, resulting in aberrant mTORC1 activation [186].

αSyn-mediated lysosomal dysfunction is further worsened by impaired membrane repair. αSyn fibrils directly target the ESCRT-III machinery, binding components such as CHMP2B, CHMP2A, and CHMP3 via conserved α-helical motifs in ESCRT-III subunits [187] and the C-terminal region (residues 103–114) of αSyn [188]. Under normal conditions, ESCRT-III directs endocytosed αSyn toward degradation via multivesicular bodies [189, 190]. Disruption of ESCRT components (reduced CHMP2B and CHMP3, increased CHMP4B/C) in Lewy body disease and αSyn models impairs this pathway, contributing to lysosomal instability and secondary deacidification [189]. In response to lysosomal stress, compensatory pathways are activated. One involves recruitment of LRRK2 to damaged lysosomes via the V-ATPase-ATG16L1 axis, promoting ATG8/LC3 lipidation on single membranes (CASM [conjugation of ATG8 to single membranes] pathway) [191, 192]. Disruption of proton gradients, a feature of αSyn pathology, acts as a trigger for V-ATPase-dependent LC3 recruitment [193]. Genetic models further support the link between V-ATPase regulation and lysosomal dysfunction. In *Drosophila*, loss of auxilin (the homolog of human GAK [cyclin G-associated kinase]) leads to lysosomal αSyn accumulation and dopaminergic neurodegeneration. Auxilin regulates phosphorylation of the V-ATPase subunit Vha44 (V1C), which is required for proper lysosomal acidification. Expression of functional Vha44 restores lysosomal acidity and rescues neurodegenerative phenotypes, highlighting a conserved pathway linking V-ATPase regulation to αSyn toxicity [194].

The extent and nature of lysosomal dysfunction are influenced by the conformational state of αSyn [48, 52, 178, 179]. Aggregated species, including oligomeric and fibrillar αSyn, are generally considered more cytotoxic than monomeric forms [48, 52]. Oligomeric αSyn preferentially associates with lysosomal membranes, either through direct interactions with lipid bilayers or through engagement with ESCRT-III components, leading to membrane destabilization and impaired ESCRT-dependent repair mechanisms [187, 188]. In contrast, fibrillar αSyn has been shown to interact with V-ATPase subunits and disrupt aspects of V-ATPase assembly or function [48]. Fibrillar αSyn species traffic through the endolysosomal pathway and can be degraded by lysosomes. However, in neurons, internalized fibrils are inefficiently cleared and can persist for extended periods, in part because αSyn aggregates induce lysosomal dysfunction. This inefficient processing can generate aggregation-prone truncated αSyn species and further exacerbate proteostatic stress [195]. Although these studies identify distinct interactions between αSyn species and lysosomal membranes, ESCRT machinery, and V-ATPase, they do not establish whether the IDRs of αSyn directly mediate these interactions. Rather, the interactions may depend on conformational states, membrane-associated structures, or aggregation-dependent interfaces. These observations suggest that distinct αSyn conformers affect lysosomal integrity and function through different mechanisms, rather than through a single conformation-independent pathway.

## Therapeutic interventions to restore lysosomal function

The preceding sections indicate that IDPs impair lysosomal acidification through both shared and distinct mechanisms. Aβ, APP-βCTF, tau, and αSyn have all been reported to interfere with V-ATPase function, either by interacting with its subunits and affecting assembly or by reducing its expression, thereby compromising proton pumping and luminal acidification. At the same time, these proteins exert additional, protein-specific effects. Aβ, APP-βCTF, and tau primarily disrupt V-ATPase-dependent acidification, whereas αSyn also affects lysosomal membrane integrity, hydrolase trafficking and activity, and membrane repair pathways. Within this context, lysosomal deacidification can be viewed not only as a downstream consequence of IDP accumulation but also as a factor that may further promote protein aggregation, linking proteostasis failure with lysosomal dysfunction. Therapeutically, this suggests two complementary strategies: reducing pathological IDP species to relieve lysosomal burden or enhancing lysosomal function to restore degradative capacity. These approaches support combining IDP-targeting and lysosome-targeting interventions, including small molecules, nanoparticles (NPs), gene-based strategies, and targeted protein degradation, to mitigate neurodegenerative disease progression.

### Small-molecule therapeutic agents

**Fig. 5:**
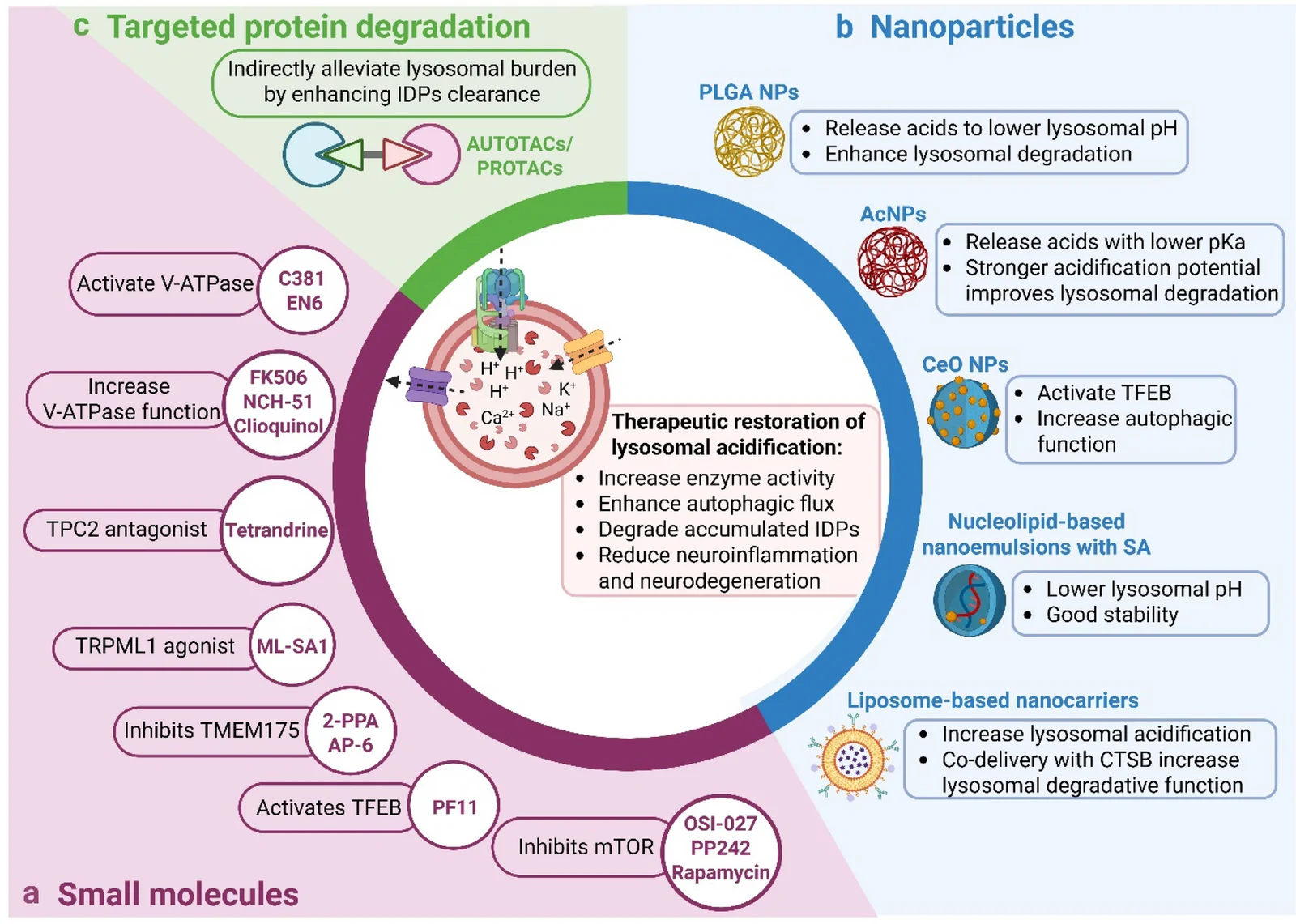
Schematic overview of therapeutic strategies targeting lysosomal dysfunction in neurodegenerative diseases. The lysosome is depicted as a central therapeutic target, with emerging approaches broadly categorized into **a** small molecules, **b** nanoparticle-based systems, and **c** targeted protein degradation strategies. These approaches act through complementary mechanisms, including restoration of lysosomal acidification and function, enhancement of lysosomal degradative capacity, and reduction of pathogenic protein burden to facilitate proteostatic clearance. PLGA: poly(lactic-co-glycolic acid); NP: nanoparticle; AcNP: acidic nanoparticle; CeO: cerium oxide; SA: succinic acid. Created with BioRender.com

Small molecules are low-molecular-weight compounds that modulate biological processes by engaging specific membrane or intracellular targets. While it is possible to directly alter IDP conformations to modulate their interactions with other molecular partners including the V-ATPase complex, these IDP-targeting small molecules that alter aggregation, conformation, or propagation have already been reviewed elsewhere [196–198]. To our knowledge, there are currently no known small molecules that have been developed to disrupt interactions between IDPs and the V-ATPase complex. Instead, most reported compounds act by modulating V-ATPase activity or lysosomal pH. Accordingly, this section focuses on summarizing small molecules that restore lysosomal acidification and function, thereby enhancing the clearance of accumulated IDPs (Fig. 5a).

Among the direct lysosomal acidification modulators are compounds that act on V-ATPase complex or its associated machinery. For example, C381 directly activates V-ATPase, enhancing proton-pumping efficiency, restoring lysosomal acidification, reactivating lysosomal hydrolases, reducing neuroinflammation, and enhancing clearance of toxic aggregates [199]. Similarly, EN6 targets the V1A subunit of V-ATPase by binding cysteine 277, thereby improving proton transport and facilitating autophagic clearance [200]. FK506 also binds ATP6V1A and exerts neuroprotective effects by promoting TFEB activation and enhancing V-ATPase activity, whereas NCH-51, a histone deacetylase inhibitor, selectively upregulates ATP6V1A expression [201], thus rescuing neuronal dysfunction and neurodegeneration. In PD, the *LRRK2*-R1441C mutation disrupts the LRRK2–ATP6V1A interaction, causing V-ATPase mislocalization, elevated lysosomal pH, and impaired autophagosome-lysosome fusion [202]. Clioquinol has further been shown to restore lysosomal pH, modulate lysosomal Zn or Cu homeostasis, and upregulate the V-ATPase A1 subunit in *LRRK2*-R1441C primary cortical neurons [202]. Clioquinol treatment also significantly attenuated dopaminergic neurodegeneration and suppressed αSyn-associated pathology in mice injected with Lewy bodies [203]. With recent advances in LRRK2 peptide modulators [204], there is potential to stabilize this interaction with peptidomimetics that can restore lysosomal acidification and counteract PD-associated dysfunction. Other small molecules improve lysosomal acidification and function by modulating lysosomal ion channels or upstream signaling pathways. Tetrandrine inhibits TPC2 ion channels, enhancing lysosomal acidification and reducing tau pathology [205]. ML-SA1 activates the lysosomal Ca^2+^ channel TRPML1, promoting Ca^2+^ release, TFEB activation, and enhanced autophagy-lysosomal clearance [206]. TMEM175-selective inhibitors, 2-phenylpyridin-4-ylamine and AP-6, have been shown to increase the level of lysosomal degradative activity [207]. Pseudoginsenoside-F11, a naturally derived ocotillol-type saponin, also enhances Aβ clearance by promoting lysosomal biogenesis, acidification, and endosomal maturation, particularly in microglia, by driving TFEB nuclear translocation and increasing expression of lysosomal genes, including V-ATPase components [208]. Finally, several compounds act primarily by enhancing autophagy rather than directly targeting lysosomal pH, including rapamycin, which inhibits mTORC1 and promotes autophagic flux for clearance of damaged organelles and protein aggregates such as tau and Aβ [209], as well as OSI-027 and PP242, which indirectly support lysosomal function through mTOR pathway inhibition [210]. These molecules illustrate multiple entry points for restoring lysosomal function, ranging from direct V-ATPase activation and ion channel modulation to broader autophagy enhancement.

### NP-based systems

As NP-based strategies that directly inhibit IDP aggregation and propagation have been comprehensively reviewed elsewhere [211], we highlight here emerging NP approaches for restoring lysosomal acidification and function (Fig. 5b). Poly(lactic-co-glycolic acid) (PLGA) NPs, for instance, are widely used for intracellular delivery of therapeutic cargos, including enzymes and small molecules, with preferential trafficking to lysosomes [212–215]. After endocytosis, PLGA NPs are delivered to lysosomes where they gradually degrade in the acidic environment, releasing lactic and glycolic acids that can contribute to local acidification and partially restore lysosomal function [212–214]. This enables improved autophagic flux and has shown therapeutic potential in various cellular and mouse models of PD [212–214]. Recently, our group has developed a new type of lysosome acidifying nanoparticles (AcNPs), engineered from poly(ethylene tetrafluorosuccinate-co-succinate) [216, 217] or poly(butylene tetrafluorosuccinate-co-succinate) [218], incorporating tetrafluorosuccinic acid, which has a low pKa and confers strong intrinsic acidity. AcNPs have demonstrated therapeutic efficacy in an APP^NL−G−F^ mouse model of AD by enhancing lysosomal function and reducing necrosome accumulation, thereby preventing neuronal necroptosis [96]. In PD, AcNPs restore lysosomal pH, enhance autophagic clearance of A30P αSyn in SH-SY5Y cells, and rescue dopaminergic neuron loss and locomotor deficits in *Drosophila* expressing A30P αSyn [217]. AcNPs have also been shown to rescue *GBA1*-associated defects in lysosomal acidification and V-ATPase assembly, thereby restoring mitophagy, mitochondrial membrane potential, and mitochondrial oxidative phosphorylation in *GBA1* mutant dopaminergic neurons [219]. Comparatively, AcNPs facilitate more efficient lysosomal reacidification than PLGA-based systems, potentially because of their lower pKa and more favorable degradation kinetics. Further studies in diverse cell types and mouse models will be necessary to validate these findings and confirm the underlying mechanisms.

Beyond polymer-based NPs, additional nanomaterial platforms further reinforce this therapeutic axis. Cerium oxide NPs activate TFEB in fibroblasts derived from patients with late-infantile neuronal ceroid lipofuscinosis, thereby enhancing autophagy, promoting aggregate clearance, and reducing oxidative stress [220]. Nucleolipid-based nanoemulsions carrying succinic-acid-derived prodrugs restore lysosomal acidification in ATP13A2-depleted neuronal cells, while showing good colloidal stability and low cytotoxicity under the tested conditions [221]. Liposome-based lysosomal pH-modulating nanocarriers similarly restore lysosomal function by re-acidifying the lysosomal lumen, thereby rescuing pH-dependent proteolytic activity and enhancing the clearance of protein aggregates. In addition, co-delivery with CTSB produces a synergistic effect, further improving lysosomal acidification and degradative capacity [222]. These nanomaterial-based strategies extend beyond passive clearance of accumulated IDPs by functionally restoring lysosomal acidification and enzymatic activity, thereby mitigating upstream drivers of IDP accumulation.

### Emerging molecular targets and other therapeutic strategies

In addition to small molecules and NPs, emerging molecular targets could enable the development of gene-based interventions to restore lysosomal functions. Among the most relevant regulators, TMEM175 is a lysosomal K^+^ channel essential for maintaining lysosomal membrane potential and pH stability, and its dysfunction has been linked to impaired autophagic degradation and αSyn accumulation in PD [166, 223, 224]. SLC7A11, a cystine/glutamate antiporter, regulates lysosomal pH through cysteine/glutamate flux across the lysosome membrane that results in slow-leaking proton movement. Loss of SLC7A11 results in excessive lysosomal acidification, with growing evidence implicating its activity in lysosomal integrity and neurodegeneration susceptibility [13]. NDST3 has been associated with regulation of acidification through involvement in lysosomal V-ATPase assembly and deacetylation of microtubules, influencing the trafficking and assembly of lysosomes [14, 225]. CLN3, a lysosomal membrane protein mutated in Batten disease, plays an important role in lysosomal pH regulation, enzyme trafficking, and overall lysosomal function. Dysfunctional CLN3 causes marked lysosomal storage and acidification defects [226, 227]. In contrast, GSK-3 regulates lysosomal and autophagic pathways indirectly through TFEB- and mTOR-dependent signaling networks, thereby modulating lysosomal biogenesis and function rather than directly affecting lysosomal ion homeostasis [228, 229]. Future advances in genome editing and gene-based therapies could offer a multifaceted strategy to restore lysosomal function and enhance degradation of pathological protein aggregates.

Another emerging strategy is targeted protein degradation, including proteolysis-targeting chimeras (PROTACs), autophagy-targeting chimeras (AUTOTACs), and lysosome-targeting chimeras (LYTACs) (Fig. 5c). PROTACs recruit E3 ligases to intracellular pathogenic proteins, promoting ubiquitination and degradation via the proteasome and thereby reducing the burden of aggregation-prone species such as αSyn, tau, and TDP-43 [230–232]. In parallel, AUTOTACs are designed to recruit p62/SQSTM1 and direct aggregation-prone intracellular proteins into the autophagy-lysosome pathway. This platform has been shown to promote lysosomal degradation of mutant huntingtin and phosphorylated tau in neurodegeneration models [233] and has also been extended to αSyn aggregates [234]. In contrast, LYTACs are optimized for extracellular and membrane-associated proteins, using cell-surface lysosome-shuttling receptors to route targets into the lysosome for degradation. Consequently, they are better suited for targeting non-cytosolic disease-associated proteins than intracellular aggregates, broadening the scope of lysosome-mediated protein clearance beyond conventional intracellular degraders [235, 236]. In sum, these platforms illustrate how targeted degradation strategies can complement lysosome-restoring approaches by facilitating the selective delivery of pathological proteins to lysosomes, while lysosome-restoring therapies re-establish the degradative capacity required for their efficient clearance.

Despite their therapeutic potential, overcoming key translational challenges will be essential for the successful clinical implementation of these approaches. One longstanding obstacle is the blood-brain barrier (BBB), which severely limits the delivery of many therapeutics to the brain, often preventing them from attaining therapeutically effective concentrations [237, 238]. In addition, evidence for lysosome-targeted therapies remains largely confined to cell and animal models, with limited clinical evaluation in neurodegenerative diseases [239]. Another concern is that restoring lysosomal acidification alone may not fully resolve upstream autophagy defects. If autophagosome formation or cargo delivery remains impaired, cells may continue to accumulate undegraded material and experience persistent proteostatic stress. Thus, while these strategies hold substantial promise for restoring lysosomal function and improving disease outcomes, further optimization to enhance BBB penetration, improve pathway specificity, and facilitate clinical translation will be necessary before their broad application in patients with neurodegenerative diseases.

## Summary and future perspectives

This review highlights the molecular interplay between IDPs, particularly Aβ, tau, and αSyn, and key regulators of lysosomal function. While these proteins converge on the disruption of V-ATPase-dependent lysosomal acidification through direct interactions with its subunits or by perturbing its assembly, they also exert distinct, protein-specific effects. Aβ, APP-βCTF, and tau primarily impair proton pump function and endolysosomal trafficking, whereas αSyn additionally compromises lysosomal membrane integrity, disrupts degradative capacity, and interferes with membrane repair pathways (Table 1). Emerging evidence suggests that other IDPs implicated in neurodegeneration, including huntingtin and TDP-43, may disrupt lysosomal function and acidification via similar mechanisms. These direct and indirect processes and downstream effects converge on the disruption of lysosomal function, with V-ATPase impairment representing a central but not exclusive node, ultimately contributing to disease progression. These perturbations lead to lysosomal alkalinization, defective degradation, and toxic protein accumulation. More broadly, these findings are consistent with an emerging metabolic-inflammatory axis, in which lysosomal dysfunction links altered cellular metabolism to the modulation of inflammatory signaling pathways [55, 240–242]. However, IDPs are not restricted to the central nervous system, as several disease-associated IDPs are also detected in peripheral tissues and circulation [243]. Whether and how peripheral IDPs influence brain lysosomal function, either through direct effects on peripheral-central communication or through a broader body-brain axis, remains an important and largely unexplored question [243–246]. Understanding these systemic interactions may reveal additional mechanisms linking peripheral proteostasis failure to neuronal vulnerability and neurodegenerative disease progression.

**Table 1:** Mechanisms by which intrinsically disordered proteins (IDPs) disrupt lysosomal and autophagic function in neurodegenerative diseases

Mechanism	Type of IDPs
Lysosome membrane destabilization or permeabilization	Aβ [39, 49] Tau [38, 50] αSyn [51–53]
Impairment of lysosomal enzyme function	Aβ [88] Tau [38] αSyn [170–172, 178, 179]
Dysregulation of V-ATPase function	Aβ [7, 10] Tau [7, 129–135] αSyn [48, 177]
mTORC1 signaling dysregulation	Aβ [93, 94] Tau [6] αSyn [186]
Reduced TFEB nuclear translocation and expression	Aβ [89] Tau [139]
Impairment of autophagy initiation or autophagosome formation	Aβ [94] Tau [6]
Impairment of autophagosome-lysosome fusion	Aβ [95] αSyn [185]
Disruption of chaperone-mediated autophagy	Tau [138] αSyn [184]
Disruption of ESCRT machinery	Aβ [92] Tau [142–145] αSyn [187]

Despite substantial progress in defining how IDPs disrupt lysosomal acidification and autophagy, several key questions remain. (1) It is still unclear whether lysosomal dysfunction represents an early pathogenic driver or arises as a secondary consequence of cellular stress, particularly across disease stages and experimental models. (2) The extent to which distinct IDP conformers (e.g., monomeric, oligomeric, and fibrillar species) differentially impact lysosomal processes, including acidification, trafficking, and degradative capacity, requires further clarification. (3) While neuronal lysosomal dysfunction has been extensively characterized, lysosomal defects in glial cells, including astrocytes and microglia, likely involve distinct and insufficiently explored mechanisms, particularly in relation to acidification, ion homeostasis, and membrane repair. (4) Additional regulators of lysosomal homeostasis, such as ion transport systems, lipid composition, and membrane repair machinery, remain incompletely understood and warrant systematic investigation. (5) Whether these mechanisms are conserved across genetic and sporadic forms of neurodegenerative disease remain unresolved. (6) Finally, despite promising preclinical advances, it is not yet established whether therapeutic restoration of lysosomal function can achieve meaningful clinical benefit in patients. Although significant translational challenges persist, recent advances in selective small-molecule design and nanotechnology-based delivery systems suggest that key technical barriers are becoming increasingly tractable. Future efforts should prioritize combinatorial and mechanism-based targeting strategies, improved precision and specificity, minimization of off-target effects, scalable delivery platforms, and rigorous validation of long-term efficacy and safety in clinically relevant models.

Looking ahead, future advances in multi-omics and spatial profiling technologies [247–249], together with functional analyses of lysosomal biology, are expected to reveal the molecular basis of lysosome heterogeneity across cell types and disease states [250, 251]. Lysosome heterogeneity at the single-organelle level can be defined by functionally diverse lysosomal subpopulations with specialized roles in protein degradation, signaling, metabolism, trafficking, membrane repair, quality control, and gene regulation [252, 253]. Resolving this heterogeneity within and between neurons, microglia, and astrocytes will facilitate the identification of cell- and organelle-specific biomarkers, therapeutic vulnerabilities, and molecular signatures predictive of disease progression and treatment response, enabling precision targeting of distinct lysosomal subpopulations. Longitudinal integration of spatial multi-omics with functional studies may further define dynamic lysosomal states throughout neurodegeneration and identify optimal therapeutic windows. Ultimately, such insights will accelerate the development of personalized therapeutic strategies tailored to lysosomal perturbations and alterations underlying specific IDP-driven neurodegenerative pathologies.

## Data Availability

Not applicable.

## Abbreviations

Aβ: Amyloid-beta
AcNPs: Acidic nanoparticles
AD: Alzheimer’s disease
ALP: Autophagy-lysosomal pathway
APP: Amyloid precursor protein
APP-βCTF: Amyloid precursor protein beta C-terminal fragment
ATP6AP1/ATP6AP2: ATPase H^+^ transporting accessory protein 1/2
ATP6V0A1/ATP6V0C/ATP6V0D1/ATP6V0D2: V-ATPase V0 domain subunits a1, c, d1, and d2
ATP6V1A/ATP6V1B2/ATP6V1D/ATP6V1E1/ATP6V1G1/ATP6V1H: V-ATPase V1 domain subunits A, B2, D, E1, G1, and H
αSyn: Alpha-synuclein
AUTOTACs: Autophagy-targeting chimeras
BBB: Blood–brain barrier
CHMP: Charged multivesicular body protein
CMA: Chaperone-mediated autophagy
CTSB/CTSD/CTSL: Cathepsin B/D/L
ER: Endoplasmic reticulum
ESCRT: Endosomal sorting complex required for transport
FTD: Frontotemporal dementia
GBA1: Glucosylceramidase beta 1
Hsc70: Heat shock cognate 70
IDPs: Intrinsically disordered proteins
IDRs: Intrinsically disordered regions
LAMP1/LAMP2A: Lysosome-associated membrane protein 1/2A
LMP: Lysosomal membrane permeabilization
LRRK2: Leucine-rich repeat kinase 2
LYTACs: Lysosome-targeting chimeras
MAPT: Microtubule-associated protein tau
mTORC1: Mechanistic target of rapamycin complex 1
MVB: Multivesicular body
NAC: Non-amyloid component
NDST3: N-deacetylase/N-sulfotransferase 3
NFTs: Neurofibrillary tangles
NPs: Nanoparticles
PD: Parkinson’s disease
PLGA: Poly(lactic-co-glycolic acid)
PROTACs: Proteolysis-targeting chimeras
PTMs: Post-translational modifications
SLC7A11: Solute carrier family 7 member 11
SNARE: Soluble N-ethylmaleimide-sensitive factor attachment protein receptor
TFEB: Transcription factor EB
TMEM175: Transmembrane protein 175
TPC2: Two-pore channel 2
TRPML1: Transient receptor potential mucolipin 1
V-ATPase: Vacuolar-type H^+^-ATPase
